# A Simple Electrostatic Precipitator for Trapping Virus Particles Spread via Droplet Transmission

**DOI:** 10.3390/ijerph18094934

**Published:** 2021-05-06

**Authors:** Koji Kakutani, Yoshinori Matsuda, Teruo Nonomura, Yoshihiro Takikawa, Takeshi Takami, Hideyoshi Toyoda

**Affiliations:** 1 Pharmaceutical Research and Technology Institute, Kindai University, Osaka 577-8502, Japan; kakutani@kindai.ac.jp; 2 Laboratory of Phytoprotection Science and Technology, Faculty of Agriculture, Kindai University, Nara 631-8505, Japan; Nonomura@nara.kindai.ac.jp; 3 Plant Center, Institute of Advanced Technology, Kindai University, Wakayama 642-0017, Japan; takikawa@waka.kindai.ac.jp; 4 Department of Internal Medicine, Clinic Jingumae, Nara 634-0804, Japan; takami66@m5.kcn.ne.jp; 5 Research Association of Electric Field Screen Supporters, Nara 631-8505, Japan; toyoda@nara.kindai.ac.jp

**Keywords:** COVID-19 virus, bacteriophage φ6, electric field, negative ions, ionic wind, respiratory droplet, aerosol, FITC, ozone bubbling

## Abstract

The purpose of this study was to develop a simple electrostatic apparatus to precipitate virus particles spread via droplet transmission, which is especially significant in the context of the recent coronavirus disease 2019 (COVID-19) pandemic. The bacteriophage φ6 of *Pseudomonas syringae* was used as a model of the COVID-19 virus because of its similar structure and safety in experiments. The apparatus consisted of a spiked, perforated stainless plate (S-PSP) linked to a direct-current voltage generator to supply negative charge to the spike tips and a vessel with water (G-water) linked to a ground line. The S-PSP and G-water surface were paralleled at a definite interval. Negative charge supplied to the spike tips positively polarised the G-water by electrostatic induction to form an electric field between them in which ionic wind and negative ions were generated. Bacteriophage-containing water was atomised with a nebuliser and introduced into the electric field. The mist particles were ionised by the negative ions and attracted to the opposite pole (G-water). This apparatus demonstrated a prominent ability to capture phage-containing mist particles of the same sizes as respiratory droplets and aerosols regardless of the phage concentration of the mist particles. The trapped phages were successfully sterilised using ozone bubbling. Thus, the present study provides an effective system for eliminating droplet transmission of viral pathogens from public spaces.

## 1. Introduction

In the context of the recent coronavirus disease 2019 (COVID-19) pandemic, the World Health Organization (WHO) has continuously emphasised the utmost importance of environmental cleaning and disinfection, as well as the importance of hand hygiene, respiratory etiquette, physical distance and avoidance of close, unprotected contact with people with a fever or respiratory symptoms [1]. Respiratory infections can be transmitted through droplets of various sizes. Droplets > 5–10 µm in diameter are referred to as respiratory droplets, while those < 5 µm in diameter are referred to as aerosols (droplet nuclei) [2]. Recent studies [3,4,5,6] have reported that the COVID-19 virus is primarily transmitted between people through respiratory droplets and contact routes, rather than airborne transmission of the pathogen. Droplets can form when a person coughs, sneezes or speaks. Therefore, it is especially important to trap these respiratory droplets containing the pathogen. 

Electric field-generation approaches have provided breakthrough techniques for controlling biotic and abiotic airborne nuisances causing public health problems. These nuisances have included infectious particles containing viruses [7,8,9], bacteria [9,10,11] and fungal spores [10,11,12,13], pollen grains that cause pollenosis [14], small flying insect pests that pass through a conventional insect net [15,16], some allergens [9], and tobacco smoke particles that can be inhaled passively [17]. All the works mentioned above presented methods for directly trapping targets in the electric field. However, the idea proposed in the present study is to precipitate mist particles carrying microbial pathogens based on the supposition of trapping infectious respiratory droplets. 

Fluid samples containing viruses and bacteria can be atomised into mist particles with a proper nebuliser [9,10,18,19]. In the present study, a nebuliser producing mist particles with a mass median diameter (MMD) of 5 µm was used to atomise water or microbial samples. The size range of the produced mist particles was between 1 and 10 µm, and more than 50% of the produced particles had a diameter of ~5 µm. This size range corresponds with the sizes of respiratory droplets (5–10 µm) and aerosols (≤5 µm) [2]. 

In this study, the virulent bacteriophage (phage) φ6 of *Pseudomonas syringae* var. *syringae* was used as a model of the COVID-19 virus because this phage possesses a similar hexagonal icosahedral structure with a lipid envelope and spikes [20]. The study proposes a corona discharge-generating apparatus (CDA) consisting of a spiked perforated metal plate linked to a voltage generator and a vessel with grounded water, which can completely capture mist particles that are introduced into the corona discharge-generating electric field of the apparatus. The apparatus can be constructed with common, inexpensive materials, and this economy is exceedingly important, especially when targeting broad-based use in economically developing nations. 

## 2. Materials and Methods 

### 2.1. Bacteriophage 

Virulent phage φ6 (NBRC105899) and host bacterium *P. syringae* var. *syringae* (MAFF810047) were purchased from the National Institute of Technology and Evaluation (Tokyo, Japan). The phage was multiplied through lytic infection into host bacterial cells, polyethylene glycol-precipitated, and purified by centrifugation procedures according to a previously described method [20]. The final pellet of phage particles was dissolved in sterilised water and differently diluted to determine the plaque forming units (PFUs) per millilitre by the double-layered agar method [20]. Specifically, the phage solution was mixed with an overnight culture of host bacterial cells. Then, this mixture was mixed with melted agarose (0.6% *w*/*v*) and finally poured onto an agar (1.2% *w*/*v*)-solidified SSE medium [20] in a Petri dish. After incubation overnight, the plaques were counted. Phage solution was prepared at 10^6^, 10^7^, and 10^8^ PFU/mL and used for the atomisation discussed below. 

### 2.2. Construction of the CDA 

**Figure 1:**
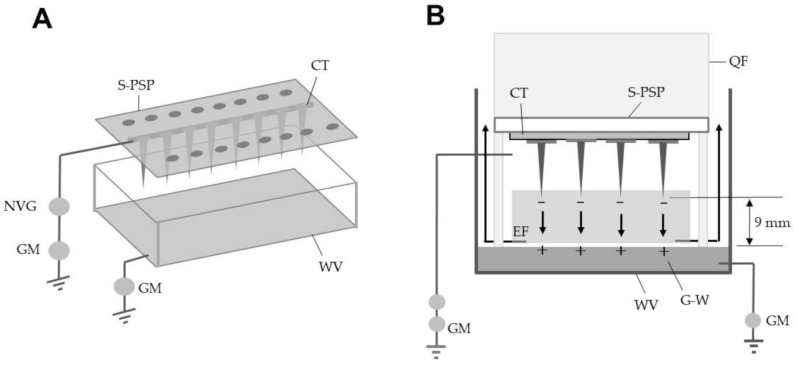
Schematic representation of the corona discharge-generating apparatus (CDA) consisting of a spiked perforated stainless plate and a water vessel (**A**) and the electric field between the spike tips and the grounded water surface (cross-sectional view) (**B**). The arrow in (**B**) represents the direction of the ionic wind produced in the electric field. S-PSP, spiked perforated stainless plate; CT, conductive double-sided adhesive tape; GM, galvanometer; NVG, negative voltage generator; EF, electric field; QF, quadrangular hood; WV, water vessel; G-W, grounded water.

A perforated stainless plate (PSP) (4 × 20 cm^2^; 0.5 mm thick) with round holes at constant intervals and a water vessel (base area, 24 × 6 cm^2^; wall height, 6 cm) were used to construct the CDA (Figure 1A). Twenty pins (pinhead diameter, 5 mm; needle length, 8 mm) were stuck to the PSP with conductive double-sided adhesive tape to construct a spiked PSP (S-PSP). The S-PSP was installed onto the open bottom end of a quadrangular hood (4 × 20 cm^2^; height, 10 cm) with four legs (Figure 1B). The CDA-installed hood was placed into the vessel with water (100 mL) (G-WT), into which the grounded line was inserted. The adhesive-tape end was connected to a direct-current voltage generator (current limit, 10 mA) (AMA-20K10NKBX1, Max-Electronics, Tokyo, Japan) and negatively charged with different voltages. Photographs of all components required to construct the CDA are presented in Figure S1A–C. The negative surface charge on the spike tips of the S-PSP causes electrostatic induction in the water, creating an opposite surface charge on the grounded water (positive electrification of the water). An electric field forms between these opposite charges (Figure 1B). The transfer of free electrons from the S-PSP side to the G-water-side ground was measured using two galvanometers (PC7000; Sanwa Electric Instrument, Tokyo, Japan) integrated into the grounded lines. 

### 2.3. Detection of Corona Discharge, Ionic Wind and Negative Ions in the Electric Field of the CDA

The corona discharge (continuous corona current) [21], which was constantly generated from the spike tips, was measured in the voltage range of −1 to −10 kV and exerted an attractive force on the mist particles. The electric current from the corona discharge was recorded with a built-in galvanometer, and the glow of the corona discharge was photographed with a long exposure in a dark field. The speed of the airflow (ionic wind) from the PSP spike tips to the G-WT generated by the corona discharge (Figure 1B) was measured at the upper outlet of the apparatus using a high-sensitivity anemometer (Climomaster 6533; Kanomax, Tokyo, Japan), and the volumetric flow rate (m^3^/min) was calculated using the equation Q (m^3^/min) = V (m^2^) × A (m/s) × 60 (s). The number of negative ions involved in the airflow was estimated with a Gerdien atmospheric ion counter (NKMH-103; Hokuto Electronic, Hyogo, Japan) [22] placed outside the outlet. 

### 2.4. Capture of Atomised Viral Sample with the CDA

In the present experiment, a compressor nebuliser (atomiser) (NE-C-28; Omron Corporation, Kyoto, Japan) (capacity of built-in water reservoir, 7 mL; rate of mist generation, 0.35 mL/min; MMD, 5 µm) [23] was used to atomise fluid samples containing fluorescein isothiocyanate (FITC) (Fuji Film Wako Pure Chemical Co., Osaka, Japan) or phage particles. The nebuliser nozzle was set at 20 cm above the CDA, and the mist was ejected towards the apparatus in the quadrangular hood. 

In the first experiment, the FITC–water (100 µg/mL) was continuously ejected toward the non-charged CDA to visually confirm that the mist particles rebounded from the water surface and were carried out of the apparatus by the force of ejection. Next, the CDA was negatively charged with different voltages (−1 to −10 kV), and the FITC–water was ejected for 30 s to determine the volumes of mist particles that were trapped by the G-WT (i.e., amounts of FITC transferred from mist particles to the G-WT) at each applied voltage. The amount of FITC in the G-water was estimated from the optical density-based calibration line formed beforehand. Trapping of FITC-mist was video-recorded under an illumination of blue-emitting diode (ASAHI ELECTRIC, Osaka, Japan). 

In the second experiment, the phage sample (10^5^–10^7^ PFU/mL) was ejected into the CDA, which was charged with different voltages (−3 to −10 kV), for 1 min. The amounts of phage particles integrated into the G-WT were determined by the double-layered agar method mentioned above. 

### 2.5. Construction of an Ozone Generator (OG) and Assay for Ozone Production 

**Figure 2:**
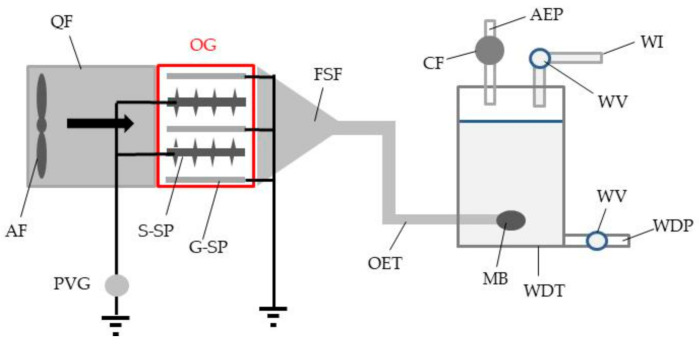
Schematic representation of an ozone generator (OG) devised for sterilising phages trapped in the grounded water. The arrow represents the direction of wind from AF. PCC, polyvinyl chloride cylinder; AF, axial fan; PVG, positive voltage generator; S-SP, spiked stainless plate; G-SP, grounded stainless plate; FSF, funnel-shaped pipe fitting; OET, ozone-ejecting tube; CF, activated charcoal filter; AEP, air exhaust port; WV, water valve; WI, water inlet; MB, micro-bubbler; WDT, water-drain tank; WDP; water-drain port.

Figure 2 shows the structure of the OG produced in the present study. The OG was constructed using two identical stainless plates (10 × 20 cm^2^; 2 mm thick). One was a spiked plate (S-SP), to which 50 metal pins were attached with conductive adhesive tape to each side and linked to a DC positive voltage generator (current limit, 10 mA) (AMF-10K1PNX2/100, Max-Electronics, Tokyo, Japan), and the other was a grounded plate (G-SP). Two S-SPs and three G-SPs were alternately and horizontally placed at intervals of 17 mm (9-mm interval between the spike tips and the G-SP). These plates were fixed with a polypropylene frame and placed inside a transparent acrylic cylinder (10 cm diameter; 30 cm length). One lateral side of the cylinder was connected to a same-sized polyvinyl chloride cylinder, whose opposite end was furnished with an axial fan, and the opposite side of the cylinder was connected to a tube (2 cm diameter; 0.5 mm thick) with a funnel-shaped pipe fitting. Photographs of all components needed to construct the OG are presented in Figure S2. The tube (ozone-ejecting tube) was connected to the flow cell of an ozone monitor (EG-700E3; Ebara Jitsugyo, Tokyo, Japan) to determine the ozone productivity. Specifically, the OG was positively charged with different voltages (−1 to −10 kV), and the air from the OG was transferred to a flow cell to measure the ultraviolet absorption at 254 nm for ozone quantification by a standard method [24]. 

In an assay for phage sterilisation, the OG was positively charged with the highest voltage (+10 kV) that caused no mechanical discharge (arc discharge) between the S-SP and G-SP, and the end of the ozone-ejecting tube was connected with a micro-bubbler (AZOO Japan, Kanagawa, Japan), which produced fine bubbles (0.5 to 20 µm diameter) and inserted into the phage-containing water (10^8^ PFU/mL) of the water drain tank (Figure 2). Exhaust from the tank was passed through an activated charcoal adsorbent to trap ozone leaked from the solution. Ozone bubbling was continued for different periods of time (5 to 40 min) to determine the optimal bubbling time for inactivating all phages in the water. After bubbling, an aliquot of the ozone-bubbled water was collected to examine the survival of the phages by the double-layered method described previously. 

### 2.6. Total CDA System for Practical Use

**Figure 3:**
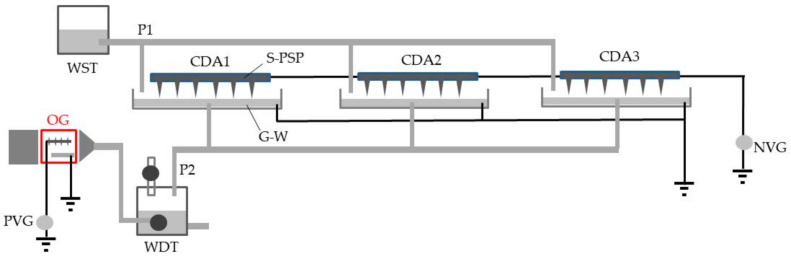
Schematic representation of the total system containing three corona discharge-generating apparatuses linked to each other (CDA1–3) and an ozone generator (OG). WST, water-supply tank; CDA1–3; three CDAs whose S-PSPs and G-waters (refer to the legend of Figure 1) are linked to a negative voltage generator and grounded line, respectively; NVG, negative voltage generator; PVG, positive voltage generator; WDT, water-drain tank; S-PSP, spiked perforated stainless plate; G-W, grounded water; P1, pipe from the water-supply tank to the water vessel of each CDA; P2, pipe from the water vessels of the CDAs to a water-drain tank.

Figure 3 shows the total system, which consists of three CDAs and a single OG. Three identical CDAs (1 m length; 50 spikes) were linked to each other and to the negative voltage generator mentioned above, and the water in the vessels was linked to a grounded line. Each water vessel was connected with two pipes; one was from a water-supply tank and the other was to a water-drain tank. The ozone-ejecting tube of the OG was connected to a micro-bubbler and inserted into a water-drain tank. The S-PSPs of the CDAs were charged with −10 kV to trap the phage-containing mist particles that were atomised, and the S-SPs of the OG were positively charged at +10 kV to generate ozone. 

Additionally, two experiments were designed to evaluate the feasibility of the present system for practical use. In the first experiment, the combined apparatuses (triplicate CDAs charged with −10 kV) were placed in a closed and unventilated cubic cabinet (side length, 2 m), and the CDAs were continuously operated (for 30 min) while and after a 5-mL phage sample (10^8^ PFU/mL) was atomised for 5 min into the air. The success of phage trapping was checked by sampling an aliquot from the water that was drained from the CDAs after the 30-min trapping operation ended. In this experiment, single and double-linked CDAs were operated similarly to compare the efficacy of the phage trapping. 

In the second experiment, the phage sample (5 mL, 10^8^ PFU/mL) was similarly ejected into the air of the cabinet, and then triple-linked CDAs (−10 kV charge) were continuously operated for 30 min after specific times (15 to 120 min) had elapsed. The water in the CDA vessels was moved to the water-drain tank to determine the number of phages obtained in each trapping operation. Phage number was determined by the method mentioned above. 

### 2.7. Statistical Analysis 

All experiments were repeated five times and data are presented as the mean and standard deviation. Analyses were performed using the EZR software version 1.54 (Jichi Medical University, Saitama, Japan) to identify significant differences among conditions and correlations among factors, which are shown in the figure and table legends. 

## 3. Results and Discussion

### 3.1. CDA Production of Negative Ions and Ionic Wind in the Electric Field 

Discharge is defined as electric current generation between opposite poles due to the dielectric breakdown of gases in an electric field according to the potential difference between the opposite poles [25]. If a grounded conductor is one of the poles (i.e., the recipient of electricity), discharge occurs more easily because this conductor receives electricity without any restriction (in this experiment, the 10-mA maximum current of the voltage generator). In the electric field, a corona discharge first occurs and then changes from a glow discharge (or surface discharge) to a brush-like discharge as the applied voltage increases and/or the distance between the poles decreases; the discharge breaks down with the occurrence of an arc discharge between the two poles [26]. The focus of the present work was the generation of glow corona discharge in the electric field, in which multiple negatively charged metallic needles can produce negative ions and an ionic wind towards the G-WT according to the principle previously reported [17]. 

In the electrostatic configuration of the present apparatus, high voltages produced through a Cockcroft circuit [27] in the voltage generator were used to pick up electricity from a ground and supply it to the spike tips of the PSP, and their negative surface charges pushed free electrons of the G-WT to the ground to create an opposite pole (see Figure 1B). In this study, we formed an electric circuit in which electricity (free electrons) moved from ground to ground when discharge of negative poles (spike tips) occurred (Figure S1D). A corona discharge occurs when the strength (potential gradient) of the electric field around a pointed metal conductor is high enough to form a conductive region but not sufficient to cause electrical breakdown or arcing to nearby objects [21]. The present electrostatic device was configured so that the charged spike tips and G-WT faced each other to produce an electric field. In this electric field, a corona discharge constantly occurred from the spike tips of the PSP to the G-WT. 

**Figure 4:**
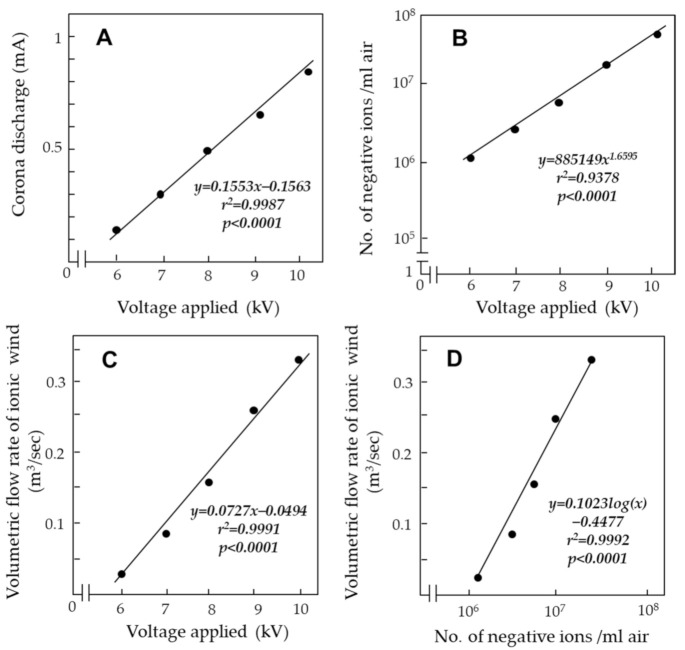
Relationship between the applied voltage and corona discharge (**A**), negative ion generation (**B**), and volumetric flow rate of ionic wind (**C**) in the electric field of the CDA. (**D**) Relationship between the volumetric flow rate of ionic wind and negative ion generation.

Figure 4A shows the relationship between the applied voltage and the corona discharge generation. In the present apparatus, the electric current produced by this discharge became greater in direct proportion to the applied voltage in the range of −6 to −10 kV. 

Measurements with an ion detector confirmed that larger voltages produced larger volumes of negative ions (Figure 4B). An ionic wind is defined as the airflow induced by electrostatic forces linked to a corona discharge arising at the tips of some sharp conductors (such as points or blades) subjected to a high voltage relative to the ground [21]. The present study showed a clear correlation between the applied voltage and generation of ionic wind—the generation increased in direct proportion to the applied voltage (Figure 4C). Figure 4D shows a positive correlation between the volumetric flow rate of ionic wind and the number of negative ions involved in the wind. The ionic wind was directed from the pointed tip of the PSP toward the G-WT, as reported in a previous work [17]. The ionic wind produced by the present apparatus was strong enough (1.5–2.0 m/s) to bring outside air into the electric field of the CDA. 

### 3.2. CDA Capture of Phage-Containing Mist Particles of the Same Size as Respiratory Droplets 

In the corona discharge-generating electric field, non-insulated conductor poles that were properly electrified could generate corona discharge against the opposite poles placed at a proper distance and secondarily generate numerous charges in the ambient air; these charges were imparted to the target for its electrification [8,9,10,11,17]. In this electric field, the electrified targets could be attracted to the opposite collection pole. In the configuration of the CDA, the G-WT acted as the positively charged collection pole (see Figure 1B). 

**Figure 5:**
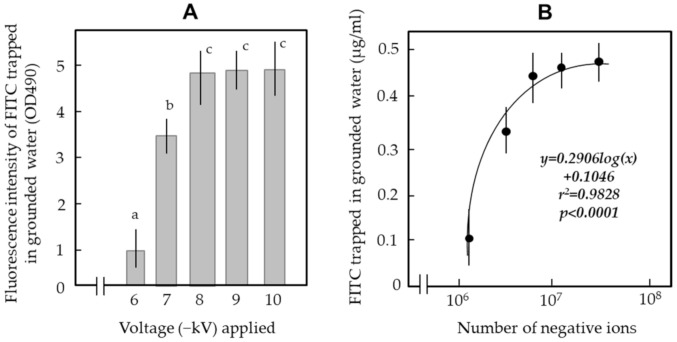
(**A**) FITC trapped in the grounded water of the CDA negatively charged with different voltages. FITC-containing mist was ejected for 30 s. The means and standard deviations were calculated from five repetitions of the experiments. The letters (a–c) on each column indicate significant differences (*p* < 0.05) according to Tukey’s method. (**B**) Positive correlation between trapping of FITC and negative ion generation in the electric field of the CDA.

In the first assay for capturing phage-free mist particles, the FITC-containing water was ejected by the atomiser. Mist particles from the atomiser could be traced visibly. In fact, the particles reached the G-WT and rebounded from its surface. This method easily and effectively determined the optimum voltage to be applied to the CDA to stop the mist rebound. Video S1 shows that the rebound of mist particles was suppressed when the voltage applied to the CDA reached −8 kV. More importantly, the amounts of FITC integrated into the water successively increased during the continuous ejection of the FITC–water into the CDA charged at this voltage (Video S2). Figure 5A shows the change in the amount of FITC integrated into the G-WT of the CDA negatively charged with different voltages. 

These results strongly indicate that the G-WT acts as a collection pole of the mist particles and that its ability to capture mist particles was enhanced in direct proportion to the voltage applied. Figure 5B shows the positive correlation between the negative ion generation and the trapped volume of FITC (i.e., mist particles) in the voltage range of −6 to −10 kV. These results strongly suggest the involvement of negative ions in the electrostatic attraction of particles in the electric field of the CDA. Thus, the results met our expectation that negative ions can ionise mist particles and thus generate an attractive force towards the opposite charge on the G-WT. 

In the second assay, the sample solution with different phage concentrations was ejected into the CDA charged in the range of −3 to −10 kV, and an aliquot (100 µL) of the G-WT was collected to determine the concentration of phage trapped in the G-WT (Table 1). The result indicated that the G-WT captured mist particles carrying phage and that the number of phages captured increased stepwise with the increase in applied voltage and reached a plateau between −8 and −10 kV. From these results, we concluded that the CDA could capture phage-containing mist particles sufficiently when charged at ≥8 kV. In addition, the present results indicate that the capture of mist particles by the CDA occurs with no relation to the concentration of phage contained in the mist particles. As described above, the capture of the mist particles depended on the surface ionisation of mist particles by negative ions, which was followed by an attraction to an opposite pole. These results suggest that the apparatus is applicable to pathogens of various bacterial (diphtheria, pertussis, meningitis, plague, pneumonia), viral (influenza, meningitis, mumps, rubella, pneumonia), and mycoplasma diseases caused by droplet transmission [28]. 

### 3.3. OG for Phage-Sterilisation Treatment Constructed with Slight Modification of the CDA 

The sterilisation of trapped pathogens is the final step of airborne pathogen precipitation. Various physical and physicochemical methods, such as use of gamma rays [29], ultraviolet radiation [30], ozone [31], plasma [32] and the application of various antimicrobial chemical reagents [33,34,35,36] have been used for this purpose. In the present study, ozone was used as a simple and inexpensive method to kill trapped pathogens because the ozone-generating apparatus could be fabricated easily with simple modification of the CDA. In fact, the OG, with its simple structure, is easy to construct and economical for practical use. The typical commercial apparatus for generating ozone is based on variations of the high-voltage electric discharge method [37,38,39,40,41,42,43,44]. Our device is based on the same physical principle. Ozone was generated effectively between the positively charged needle pole and the oppositely charged, grounded metallic plate [44]. 

Ozone, as an antimicrobial agent, effectively kills viruses, bacteria and fungi in water [37,38,39,40,42,44,45,46]. To disinfect a solution infested with pathogens, the solution must be subjected to a bubbling of ozonic air with a certain concentration of ozone and proper exposure time [41]. Runia [47] reported that a 1-h supply of 10 g of ozone is sufficient to kill all pathogens in 1 m^3^ of water. On the other hand, Vestergård [39] reported that a much lower dose of ozone is sufficient if the ozone is supplied constantly to the culture solution from the beginning of the culture. Some researchers have shown that a small bubble size is required to obtain improved microorganism–bubble contact and increase the gas–liquid interfacial area [40,42,44,45,46,48]. In our system, a micro-bubbler was used to create fine bubbles (~20 µm diameter) of ozonic air from the OG. Under the present conditions of continuous aeration, small bubbles persisted in the ozonated solution with only a slight decrease during the operation. 

**Figure 6:**
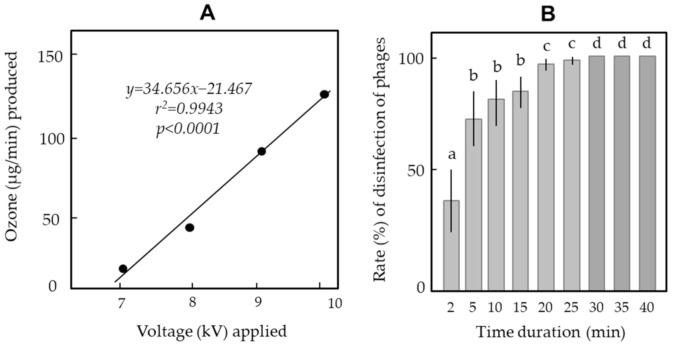
Positive correlation between ozone productivity and voltage applied to the ozone generator (**A**) and change in the fatality rate with time of phages exposed to microbubbles produced by the ozone generator (10-kV charge) (**B**). The means and standard deviations in (**B)** were calculated from five repetitions of the experiments. The letters (a–d) on each column indicate significant differences (*p* < 0.05) according to Tukey’s method.

Figure 6A shows a clear correlation between the ozone production and the voltage applied in the voltage range (+7 to +10 kV) that caused no mechanical discharge. The ozone productivity increased in direct proportion to the voltage applied in this range. In the following experiment, the OG was charged at +10 kV to inactivate phage particles in the water. Figure 6B shows the optimal ozone-bubbling time required to achieve complete sterilisation of the phages. The result indicated that 30 min of bubbling was sufficient to kill phages completely for a safe drain. 

### 3.4. Total CDA System as a Practical Tool for Trapping and Inactivating Phages in Mist Particles Sprayed into the Air 

Respiratory droplets form when a person coughs, sneezes or speaks and typically travel only short distances (usually less than 1 m) before settling. The droplets are usually too large to be airborne for long periods of time and quickly settle out of the air. According to current evidence, the COVID-19 virus is primarily transmitted by respiratory droplets and contact routes [49]. Our major concern in the present work was to determine whether the suspended mist particles could be trapped with the CDA. 

**Figure 7:**
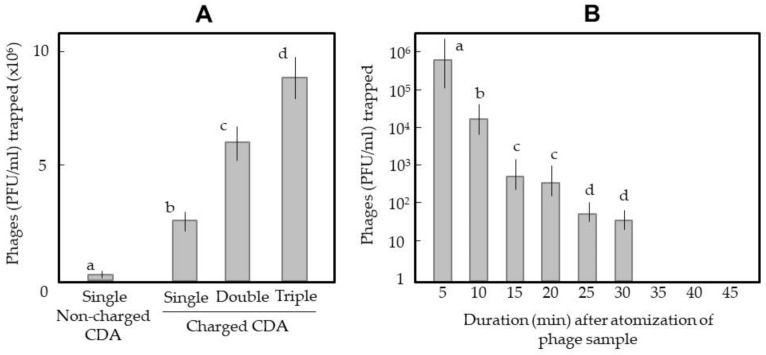
(**A**) Trapping of phages atomised into air with a single CDA and with double- and triple-linked CDAs. A non-charged CDA was used singly as a negative control. The trapping time was 30 min. (**B**) Time relationship of trapping of phages atomised into the air with the triple-linked CDAs. In both experiments, 5 mL of phage solution (10^8^ PFU/mL) was atomised for 5 min and collected for 30 min with −10 kV-charged CDA(s). Phage-trapping efficiency was determined by the double-layered agar method. The means and standard deviations in (**B**) were calculated from five repetitions of the experiments. The letters (a–d) in each graph indicate significant differences (*p* < 0.05) according to Tukey’s method.

Figure 7A shows comparative data of phage-trapping efficiency among single, double, and triple use of the CDA. The charged CDA effectively trapped phages atomised in the air. This positive result seemed to be due to the generation of ionic wind by the CDA, which was useful for drawing outside air into the CDA. In fact, the double and triple CDAs were more effective at drawing larger volumes of air, that is, larger numbers of phage particles. 

Another question was how long the atomised phage mists with the present sizes (1–10 μm) could float in the air of the non-ventilated closed cabinet. To answer this question, a time-course trapping assay was conducted using the triple-linked CDAs (Figure 7B). The number of trapped phages was highest in the first trapping operation and then decreased through time. At 35 min after atomisation and later, no phage was obtained by the trapping operation. These results suggest that the phage-containing mist particles continued to float in the air, at least in the non-ventilated cabinet.

The present study demonstrated that the CDA has a strong ability to precipitate phage particles atomised in the air. However, the suspended droplets can evaporate in seconds, depending on the temperature and relative humidity [50]. Therefore, it is likely that the pathogens ejected by the atomiser lost their mist water during suspension before and after the mist particles entered the electric field of the CDA. Although this study provided no direct evidence elucidating these possibilities, this point raised the question of whether the CDA could capture phage particles that had lost their water. In the former case, that is, initial evaporation, the problem is whether the water-deprived phage particles can be ionised negatively in the electric field. However, to this point, some researchers have noted that in a corona discharge-generating electric field, numerous negative ions are generated in the ambient air of the field and imparted to biotic (viruses, bacteria and spores) [8,9,10,11] and abiotic (fine tobacco smoke particles) targets [17] that entered the electric field. Furthermore, in the present study, negative ions were produced abundantly in the electric field of the CDA (see Figure 4B). According to the aforementioned reports, the water-deprived phages could be directly ionised with negative ions and thus be attracted to the opposite pole (in the present case, grounded water). On the other hand, in the latter case, that is, the mist particles being ionised in the electric field and then evaporated, the principle of pathogen precipitation presented in this study is to negatively ionise the mist particles in the electric field for attraction by the positively electrified conductor pole. According to Dole’s hypothesis, which states that a droplet’s charge remains unchanged as it evaporates [51], the negative charge given to the mist particles could be retained on the surface of the pathogens originally contained in the mist particle. This retained charge on the pathogen surface could be useful for attracting it to the collection pole. This problem will be analysed further in a subsequent work.

In our preliminary work, we confirmed that the present apparatus can trap not only a membranous phage just like the φ6, but also non-membranous ones such as T7 and lambda phages of *Escherichia coli*, and moreover gram-negative (*E. coli*) and positive (*Bacillus subtilis*) bacterial cells. Judging from these results, it is likely that the present apparatus can produce abundant negative ions enough to surpass the original surface charge of the targets, even if they were in any state of surface charge. Although we used the φ6 phage as a model of the COVID-19 virus because of its morphological similarity and safe use in experiment, any types of microorganisms may be applied to assess the feasibility of the present apparatus to precipitate airborne- and droplet-transmitted pathogens.

In this system, the water that included the trapped phages was successfully collected in the water-drain tank for sterilisation. Sterilisation by 30 min of ozone-bubbling treatment was sufficient to kill all phages. Thus, the present CDA system could be applicable as a practical approach for precipitating infectious respiratory droplets containing viral pathogens.

The limitation of the present apparatus was the ability to draw outside air into the electric field, which must be improved for eliminating air-floating pathogens more quickly from the space of larger aera Possible step for this purpose is to establish more pin spikes, which are useful to increases the site of corona discharge to generate stronger ionic wind; eventually the stronger ionic wind enables the entry of larger volumes of the air into the apparatus. Additionally, the attachment of the air supplier to the hood of the CDA is helpful to send the air to the apparatus. These parameters must be optimised in accordance with the space area of target.

In the present study, we demonstrated that the present apparatus has a remarkable ability to eliminate the phage particles atomized in the air. In this work, however, we obtained no direct evidence to show the ability of the present apparatus to eliminate the COVID-19 virus (SARS-CoV-2 virus) air-floating via droplet transmission, and therefore the present work is taken as a proof of concept for air-borne pathogen elimination.

### 3.5. Fabrication of the CDA and OG at Considerably Low Cost

The framework of the apparatus can be fabricated simply and easily with common metal materials. The voltage generator, which can be operated with a 12-V storage battery, is the only electrical instrument that needs to be purchased. The voltage generator can also be used to boost the initial voltage (12 V) to the designated voltage (1–30 kV) via a transformer (coil) and Cockcroft circuit integrated into an electric circuit in the voltage generator [27]. Two configurations for the voltage generator—fixed voltage and adjustable voltage—are commercially available. During our investigations, the adjustable voltage model was used to examine the relationship between the voltages applied and the occurrence of electrostatic phenomena. Ultimately, a fixed voltage model was used in later experiments due to the lower cost of this type of generator. Thus, the present study provides an experimental basis for developing an economical electrostatic precipitator to eliminate infectious respiratory droplets from a public space.

## 4. Conclusions

The electric field-generating approach provides a breakthrough technique for precipitating airborne viral pathogens traveling via droplet transmission. The electrostatic apparatus devised in this study has a simple configuration, which creates a dynamic electric field that produces an ionic wind to draw outside air into the electric field and negative ions to negatively electrify the introduced target for attraction to the oppositely charged trapping pole (grounded water). This apparatus demonstrated a strong ability to capture large volumes of mist continuously ejected from an atomiser and, more importantly, to trap the pathogen particles contained in the mist, which was equivalent in size to respiratory droplets, regardless of pathogen concentration. Accordingly, this apparatus would be effective for precipitating infectious respiratory droplets, especially in crowded and less-ventilated places. The present apparatus is easy to construct with ubiquitous materials and can be combined with various methods (in this case, ozone bubbling), if necessary, for sterilisation of the water containing the pathogens. The low production cost and low electric power consumption of the present apparatus are advantages that facilitate its use, especially in economically developing nations, for arresting the transmission of airborne pathogens.

## Figures and Tables

**Table 1:** Number of plaques produced by phage φ6 particles recovered from the grounded water of the CDA ^I^.

Voltage (−kV) Applied to the CDA	Phage Concentration (PFU/mL) Used for Atomization		
	10^5^	10^6^	10^7^
3	0 ^a^	0.4 ± 0.4 ^a^	195.2 ± 11.6 ^a^
4	0 ^a^	0.8 ± 0.50 ^a^	273.6 ± 15.8 ^b^
5	0 ^a^	5.0 ± 2.1 ^a^	327.6 ± 27.9 ^b^
6	0 ^a^	22.4 ± 7.7 ^b^	844.4 ± 88.0 ^c^
7	1.4 ± 0.6 ^b^	91.0 ± 18.9 ^c^	1030.0 ± 84.5 ^c^
8	1.5 ± 1.0 ^b^	231.2 ± 26.4 ^d^	1335.8 ± 81.4 ^d^
9	1.2 ± 0.8 ^b^	215.2 ± 13.7 ^d^	1399.2 ± 62.3 ^d^
10	1.4 ± 0.6 ^b^	258.4 ± 55.6 ^d^	1470.4 ± 42.4 ^d^

^I^ Sample solutions with different phage concentrations were separately atomised toward the CDA for 1 min, then an aliquot of the grounded water of the CDA was collected to determine the concentration of the phages trapped in the water by the double-layered agar method. The means and standard deviations were calculated from five repetitions of the experiments. The letters (a–d) on the means in each vertical column indicate significant differences (*p* < 0.05) according to Tukey’s method.

## Data Availability

Not applicable.

## Supplementary Materials

The following are available online at https://www.mdpi.com/article/10.3390/ijerph18094934/s1, Figure S1: (A) Components used to construct the corona discharge-generating apparatus (CDA). (B) A line of metal pins attached to the perforated stainless plate. (C) The quadrangular hood placed inside the water vessel with water (GW). (D) Corona discharge glow (CDG) from the tips of the pin spikes of the CDA (charged with −10 kV) photographed in a dark field, Figure S2: (A) An ozone-generating system devised for phage sterilisation. (B) An ozone generator consisting of two stainless plates (S-SP) with metal pins attached on each side and three grounded stainless plates (G-SP), Video S1: Ejection of mist particles of FITC-containing water into the CDA, Video S2: Trapping of FITC-containing mist particles ejected into the non-charged (upper) and −8 kV-charged CDA (lower).

## References

[1] World Health Organization Infection Prevention and Control (IPC) for Novel Coronavirus (COVID-19) Course. https://openwho.org/courses/COVID-19-IPC-EN.

[2] World Health Organization (2014). Infection Prevention and Control of Epidemic- and Pandemic-Prone Acute Respiratory Infections in Health Care.

[3] Liu J., Liao X., Qian S., Yuan J., Wang F., Liu Y., Wang Z., Wang F.-S., Liu L., Zhang Z. (2020). Community transmission of severe acute respiratory syndrome coronavirus 2, Shenzhen, China, 2020. Emerg. Infect. Dis..

[4] Chan J.F.-W., Yuan S., Kok K.-H., To K.K.-W., Chu H., Yang J., Xing F., Liu J., Yip C.C.-Y., Poon R.W.-S. (2020). A familial cluster of pneumonia associated with the 2019 novel coronavirus indicating person-to-person transmission: A study of a family cluster. Lancet.

[5] Li Q., Guan X., Wu P., Wang X., Zhou L., Tong Y., Ren R., Leung K.S.M., Lau E.H., Wong J.Y. (2020). Early transmission dynamics in Wuhan, China, of novel coronavirus-infected pneumonia. N. Engl. J. Med..

[6] Huang C., Wang Y., Li X., Ren L., Zhao J., Hu Y., Zhang L., Fan G., Xu J., Gu X. (2020). Clinical features of patients infected with 2019 novel coronavirus in Wuhan, China. Lancet.

[7] Hogan C.J., Lee M.H., Biswas P. (2004). Capture of viral particles in soft X-ray-enhanced corona systems: Charge distribution and transport characteristics. Aerosol Sci. Technol..

[8] Kettleson E.M., Ramaswami B., Hogan C.J., Lee M.-H., Statyukha G.A., Biswas P., Angenent L.T. (2009). Airborne virus capture and inactivation by an electrostatic particle collector. Environ. Sci. Technol..

[9] Kettleson E.M., Schriewer J.M., Buller R.M.L., Biswas P. (2013). Soft X-ray enhanced electrostatic precipitation for protection against inhalable allergens, ultrafine particles, and microbial infections. Appl. Environ. Microbiol..

[10] Li C.S., Wen Y.M. (2003). Control effectiveness of electrostatic precipitation on airborne microorganisms. Aerosol Sci. Technol..

[11] Lee S.-A., Willeke K., Mainelis G., Adhikari A., Wang H., Reponen T., Grinshpun S.A. (2004). Assessment of electrical charge on airborne microorganisms by a new bioaerosol sampling method. J. Occup. Environ. Hyg..

[12] Matsuda Y., Ikeda H., Moriura N., Tanaka N., Shimizu K., Oichi W., Nonomura T., Kakutani K., Kusakari S., Higashi K. (2006). A new spore precipitator with polarized dielectric insulators for physical control of tomato powdery mildew. Phytopathology.

[13] Takikawa Y., Matsuda Y., Nonomura T., Kakutani K., Kimbara J., Osamura K., Kusakari S., Toyoda H. (2014). Electrostatic guarding of bookshelves from mould-free preservation of valuable library books. Aerobiologia.

[14] Takikawa Y., Matsuda Y., Nonomura T., Kakutani K., Kusakari S., Toyoda H. (2017). An electrostatic-barrier-forming window that captures airborne pollen grains to prevent pollinosis. Int. J. Environ. Res. Public Health.

[15] Tanaka N., Matsuda Y., Kato E., Kokabe K., Furukawa T., Nonomura T., Honda K., Kusakari S., Imura T., Kimbara J. (2008). An electric dipolar screen with oppositely polarized insulators for excluding whiteflies from greenhouses. Crop Prot..

[16] Matsuda Y., Nonomura T., Kakutani K., Takikawa Y., Kimbara J., Kasaishi Y., Kusakari S., Toyoda H. (2011). A newly devised electric field screen for avoidance and capture of cigarette beetles and vinegar flies. Crop Prot..

[17] Matsuda Y., Takikawa Y., Nonomura T., Kakutani K., Okada K., Shibao M., Kusakari S., Miyama K., Toyoda H. (2018). A simple electrostatic device for eliminating tobacco sidestream to prevent passive smoking. Instruments.

[18] Yao M., Mainelis G., An H.R. (2005). Inactivation of microorganisms using electrostatic fields. Environ. Sci. Technol..

[19] van Doremalen N., Bushmaker T., Morris D.H., Holbrook M., Gamble A., Williamson B.N., Tamin A., Harcourt J.L., Thornburg N.J., Gerber S. (2020). Aerosol and Surface Stability of SARS-CoV-2 as compared with SARS-CoV-1. N. Engl. J. Med..

[20] Vidaver A.K., Koski R.K., Van Etten J.L. (1973). Bacteriophage φ6: A Lipid-containing virus of *Pseudomonas phaseolicola*. J. Virol..

[21] Jonassen N. (2002). Electrostatic effects and Abatement of static electricity. Electrostatics.

[22] Aplin K.L., Harrison R.G. (2000). A computer-controlled Gerdien atmospheric ion counter. Rev. Sci. Instrum..

[23] Nishimura H. (2017). Investigation of practical usefulness of body-worn devices that claim to release chlorine dioxide. Jpn. J. Infect. Prevent. Cont..

[24] Francis A.W., Geller E., Moore K., Well J., Blumet D., Felsenfeld S., Martin T., Rappaport A., Wagner C., Lai B., Taylor R. (2002). Ozone. McGraw-Hill Encyclopedia of Science and Technology.

[25] Halliday D., Resnick R., Walker J., Johnson S., Ford E. (2005). Electric discharge and electric fields. Fundamentals of Physics.

[26] Kaiser K.L., McLaughlsn J.C. (2006). Air breakdown. Electrostatic Discharge.

[27] Wegner H.E., Geller E., Moore K., Well J., Blumet D., Felsenfeld S., Martin T., Rappaport A., Wagner C., Lai B., Taylor R. (2002). Electrical charging generators. McGraw-Hill Encyclopedia of Science and Technology.

[28] Air/Droptlet Borne Disease. https://www.moh.gov.sg/docs/librariesprovider5/resources-statistics/reports/air-droplet-borne-diseases.pdf.

[29] Spire B., Barré-Sinoussi F., Dormont D., Montagnier L., Chermann J.C. (1985). Inactivation of lymphadenopathy-associated virus by heat, gamma rays, and ultraviolet. Lancet.

[30] Kowalski W.J., Bahnfleth W.P., Witham D.L., Severin B.F., Whittam T.S. (2000). Mathematical modeling of ultraviolet germicidal irradiation for air disinfection. Quant. Microbiol..

[31] Tseng C.-C., Li C.-S. (2006). Ozone for inactivation of aerosolized bacteriophages. Aerosol Sci. Technol..

[32] Xia T., Kleinheksel A., Lee E.M., Qiao Z., Wigginton K.R., Clack H.L. (2019). Inactivation of airborne viruses using a packed bed non-thermal plasma reactor. J. Phys. D: Appl. Phys..

[33] Vohra A., Goswami D.Y., Deshpande D.A., Block S.S. (2005). Enhanced photocatalytic inactivation of bacterial spores on surfaces in air. J. Ind. Microbiol. Biotechnol..

[34] Kariwa H., Fujii N., Takashima I. (2004). Inactivation of SARS coronavirus by means of povidone-iodine, physical conditions, and chemical reagents. Jpn. J. Vet. Res..

[35] Lee B.U., Yun S.H., Ji J.H., Bae G.N. (2008). Inactivation of *S. epidermidis*, *B. subtilis*, and *E. coli* bacteria bioaerosols deposited on a filter utilizing airborne silver nanoparticles. J. Microbiol. Biotechnol..

[36] Ogata N., Sakasegawa M., Miura T., Shibata T., Takigawa Y., Taura K., Taguchi K., Matsubara K., Nakahara K., Kato D. (2016). Inactivation of airborne bacteria and viruses using extremely low concentrations of chlorine dioxide gas. Pharmacology.

[37] Katzenelson E., Kletter B., Shuval H.I. (1974). Inactivation kinetics of viruses and bacteria in water by use of ozone. J. Am. Water Works Assoc..

[38] Burleson G.R., Murray T.M., Pollard M. (1975). Inactivation of viruses and bacteria by ozone, with and without sonication. Appl. Microbiol..

[39] Vestergård B. (1994). Establishing and maintaining specific pathogen free (SPF) conditions in aqueous solutions using ozone. Adv. Space Res..

[40] Khadre M.A., Yousef A.E., Kim J.-G. (2001). Microbiological aspects of ozone applications in food: A review. J. Food Sci..

[41] van Os E.A. (2001). Design of sustainable hydroponic systems in relation to environment–friendly disinfection methods. Acta Hortic..

[42] Kim J.-H., Rennecker J.L., Tomiak R.B., Marińas B.J., Miltner R.J., Owens J.H. (2002). Inactivation of Cryptosporidium Oocysts in a pilot-scale ozone bubble-diffuser contactor. II: Model validation and application. J. Environ. Eng..

[43] Takayama M., Ebihara K., Stryczewska H., Ikegami T., Gyoutoku Y., Kubo K., Tachibana M. (2006). Ozone generation by dielectric barrier discharge for soil sterilization. Thin Solid Films.

[44] Farooq S., Chian E.S., Engelbrecht R.S. (1977). Basic concepts in disinfection with ozone. J. Water Pollut. Control Fed..

[45] Haas C.N., Gould J.P. (1979). Disinfection. J. Water Pollut. Control Fed..

[46] Vanachter A., Thys L., Van Wambeke E., Van Assche C. (1988). Possible use of ozone for disinfestation of plant nutrient solutions. Acta Hortic..

[47] Runia W.T. (1995). A review of possibilities for disinfection of recirculation water from soilless cultures. Acta Hortic..

[48] Shimizu K., Matsuda Y., Nonomura T., Ikeda H., Tamura N., Kusakari S., Kimbara J., Toyoda H. (2007). Dual protection of hydroponic tomatoes from rhizosphere pathogens *Ralstonia solanacearum* and *Fusarium oxysporum* f. sp. *radicis-lycopersici* and airborne conidia of *Oidium neolycopersici* with an ozone-generative electrostatic spore precipitator. Plant Pathol..

[49] World Health Organization Modes of Transmission of Virus Causing COVID-19: Implications for IPC Precaution Recommendations. https://www.who.int/news-room/commentaries/detail/modes-of-transmission-of-virus-causing-covid-19-implications-for-ipc-precaution-recommendations.

[50] Bahl P., Doolan C., de Silva C., Chughtai A.A., Bourouiba L., MacIntyre C.R. (2020). Airborne or droplet precautions for health workers treating COVID-19?. J. Infect. Dis..

[51] Cole R.B. (2000). Some tenets pertaining to electrospray ionization mass spectrometry. J. Mass Spectrom..

