# Supplementary material for: A Simple Electrostatic Precipitator for Trapping Virus Particles Spread via Droplet Transmission
Source: Int J Environ Res Public Health. 2021 May 6;18(9):4934. doi: 10.3390/ijerph18094934 (PMC8124561; doi:10.3390/ijerph18094934)
Supplement: Supplementary file 1 [file ijerph-18-04934-s001.zip › Figure S1-S2.pdf]

Figure S1

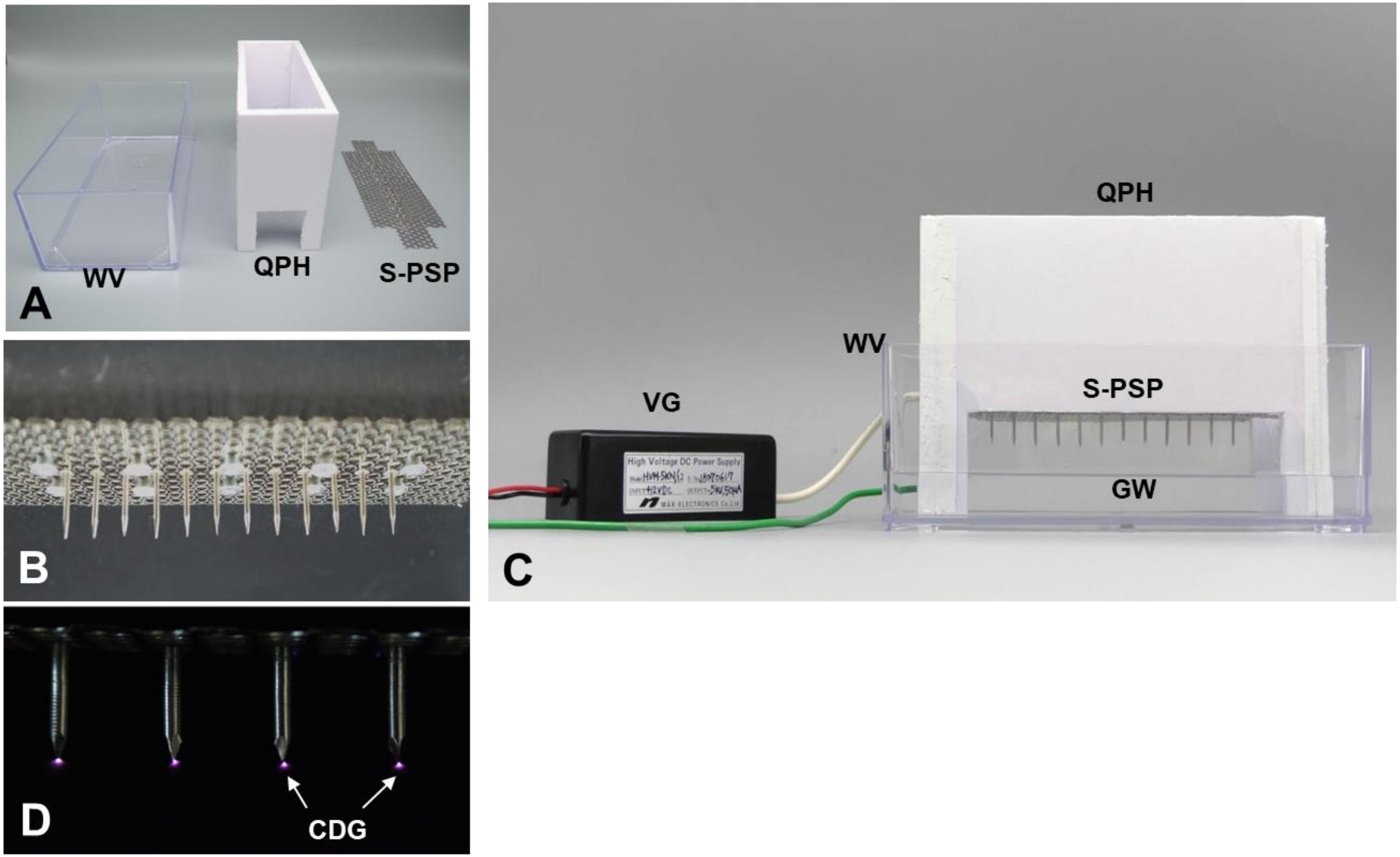

Figure S1. A) Components used to construct the corona discharge-generating apparatus (CDA): a transparent acrylic water vessel (WV), quadrangular polypropylene hood (QPH) with four legs, and a spiked, perforated stainless plate (S-PSP) (metal pins attached), which was installed at the bottom of the quadrangular hood. (B) A line of metal pins attached to the perforated stainless plate. (C) The quadrangular hood placed inside the water vessel with water (GW). The green grounded line was inserted into the vessel. The spiked, perforated stainless plate was linked to a voltage generator (VG) with the white electrical wire. (D) Corona discharge glow (CDG) from the tips of the pin spikes of the CDA (charged with −10 kV) photographed in a dark field.

Figure S2

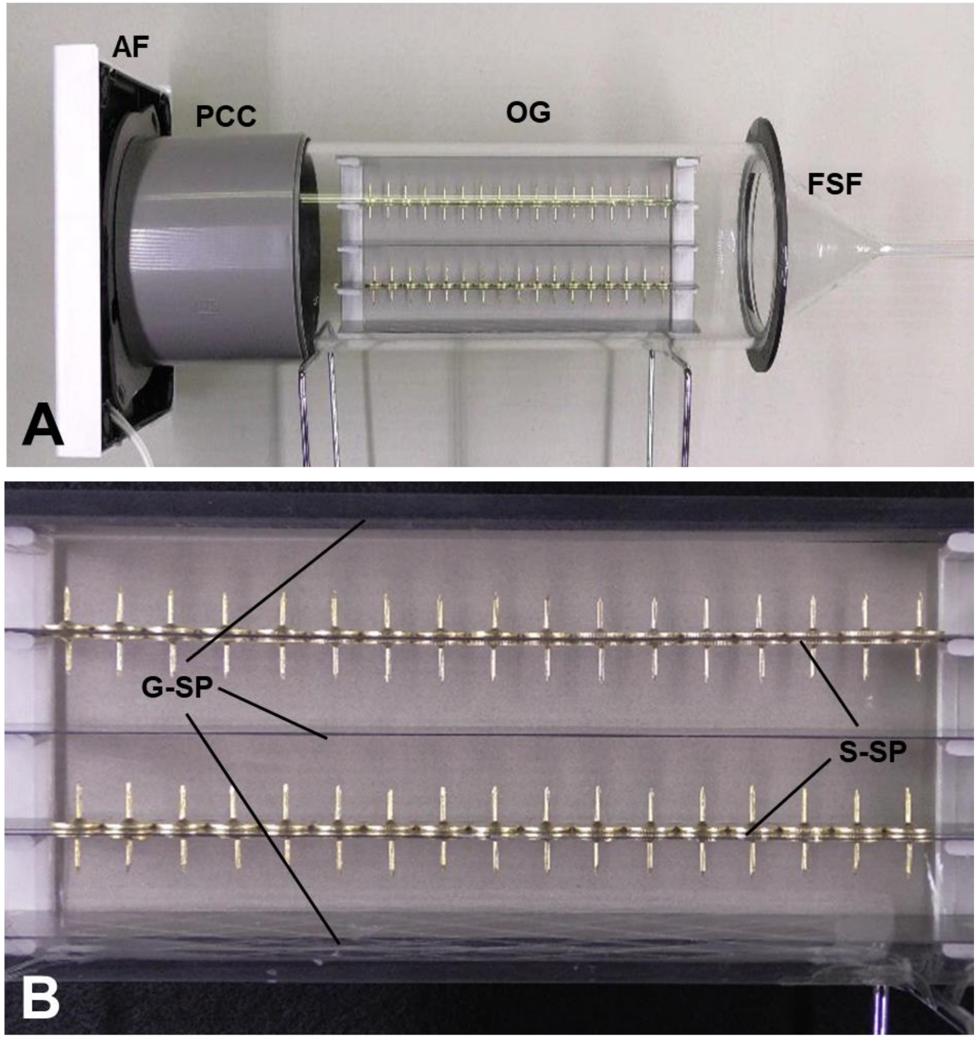

Figure S2. (A) An ozone-generating system devised for phage sterilisation. An ozone generator (OG) was placed in a transparent acrylic cylinder. One end of the OG was furnished with a funnel-shaped fitting (FSF) and the other was fitted with a polyvinyl chloride cylinder (PCC) to which an axial fan (AF) was attached. (B) An ozone generator consisting of two stainless plates (S-SP) with metal pins attached on each side and three grounded stainless plates (G-SP).
